# Optimization of the Synthesis of Superhydrophobic Carbon Nanomaterials by Chemical Vapor Deposition

**DOI:** 10.1038/s41598-018-21051-3

**Published:** 2018-02-09

**Authors:** Mustafa Mohammed Aljumaily, Mohammed Abdulhakim Alsaadi, Rasel Das, Sharifah Bee Abd Hamid, N. Awanis Hashim, Mohamed Khalid AlOmar, Haiyam Mohammed Alayan, Mikhail Novikov, Qusay F. Alsalhy, Mohd Ali Hashim

**Affiliations:** 1 Nanotechnology & Catalysis Research Centre (NANOCAT), IPS Building, University of Malaya, 50603 Kuala Lumpur, Malaysia; 2 University of Malaya Centre for Ionic Liquids, University Malaya, Kuala Lumpur, 50603 Malaysia; 3 National Chair of Materials Sciences and Metallurgy, University of Nizwa, Sultanate of Oman, Nizwa, Oman; 4 Department of Chemical Engineering, University of Malaya, Kuala Lumpur, 50603 Malaysia; 5 Department of Civil Engineering, University of Malaya, Kuala Lumpur, 50603 Malaysia; 6 Membrane Technology Research Unit, Chemical Engineering Department, University of Technology, Alsinaa Street No. 52, B. O. 35010, Baghdad, Iraq

## Abstract

Demand is increasing for superhydrophobic materials in many applications, such as membrane distillation, separation and special coating technologies. In this study, we report a chemical vapor deposition (CVD) process to fabricate superhydrophobic carbon nanomaterials (CNM) on nickel (Ni)-doped powder activated carbon (PAC). The reaction temperature, reaction time and H_2_/C_2_H_2_ gas ratio were optimized to achieve the optimum contact angle (CA) and carbon yield (CY). For the highest CY (380%) and CA (177°), the optimal reaction temperatures were 702 °C and 687 °C, respectively. However, both the reaction time (40 min) and gas ratio (1.0) were found to have similar effects on CY and CA. Based on the Field emission scanning electron microscopy and transmission electron microscopy images, the CNM could be categorized into two main groups: a) carbon spheres (CS) free carbon nanofibers (CNFs) and b) CS mixed with CNFs, which were formed at 650 and 750 °C, respectively. Raman spectroscopy and thermogravimetric analysis also support this finding. The hydrophobicity of the CNM, expressed by the CA, follows the trend of CS-mixed CNFs (CA: 177°) > CS-free CNFs (CA: 167°) > PAC/Ni (CA: 65°). This paves the way for future applications of synthesized CNM to fabricate water-repellent industrial-grade technologies.

## Introduction

Superhydrophobic carbon-based nanomaterials (CNM) with contact angles (CA) > 150° have attracted tremendous scientific and commercial interests due to their various applications, including antifouling and self-healing membranes^1^, adsorbants^2^, anti-wetting X-ray sample holders^3^, ultrasensitive protein spectroscopy substrates, microarray devices^4^, drug delivery materials^5^, and others^6,7^. Both the surface roughness and surface chemistry can affect the hydrophobicity of the materials^8^. Growing CNM on other materials, rather than altering the surface chemistry^9^, can substantially transform the surface roughness of the nanomaterial and has recently gained popularity. Nanomaterials with altering surface chemistry may not effectively acquire superhydrophobicity due to the limitations of interfacial tension^9,10^. Therefore, new and/or improved methods are necessary to synthesize carbon superstructures consisting of different hybrids that could ensure a certain surface roughness needed for superhydrophobicity.

Methods such as chemical vapor deposition (CVD), template synthesis, electrochemical deposition, crystallization control, etc., have been utilized to yield geometrical surface structures^11^, among which CVD is widely used due to its wide availability, ease of handling, and high yield^12^. An ideal substrate for CVD is powder activated carbon (PAC) due to its wide precursor availability, low cost, and potential for chemical modifications^13^. Some researchers^14–17^ have used PAC to synthesize carbon nanofibers (CNFs) using ethylene (C_2_H_4_) and iron (Fe) catalysts in the CVD processes^13,18–21^. Alternatively, some researchers have modified the structure of CNM to achieve artificial superhydrophobic surfaces. For instance, Hong *et al*. used NF_3_ plasma to decrease the surface free energy of carbon nanotubes (CNTs), which produces a CA > 150°^22^. Most CNTs aggregate and often contain impurities, which may hamper optimization of the synthesis of superhydrophobic CNM^23^. Comparatively, PAC has sizes <100 µm and offers an economical and available substrate for CNM growth^24^. Unlike other substrates, PAC does not need to be chemically or physically removed from the functional bulk material^25^. Cleaning and washing processes negatively affect the surface of CNM and dramatically reduce the CA^26^. The hybrid PAC-CNF shows the combined properties of a classical PAC and CNFs while maintaining the chemical compatibility between these two materials. However, these studies have not considered the synthesis of different CNM, and the lack of optimization for increasing the carbon yield (CY) and CA have left scientists ignorant about the process. Therefore, our efforts in this study were focused on the determination of the optimum reaction conditions for efficient production of superhydrophobic CNM.

Herein, we successfully synthesized two hierarchical superhydrophobic CNM, carbon sphere (CS)-free CNFs and CS-mixed CNFs, using acetylene (C_2_H_2_) and a nickel (Ni) catalyst in thermal CVD at 650 and 750 °C, respectively. We found that optimization of the reaction conditions, such as the reaction temperature, reaction time, and gas ratio, was important to increase both the CY and CA. Both the CS-free CNFs and CS-mixed CNFs showed CA > 150° for a 4 µL droplet. Such hierarchical structure, i.e., CS nested in CNFs incorporated in PAC, is promising for applications in sorption^2,27^, membrane distillation^28,29^, organic mixture separation, water adsorptive purification, and catalysis^30^.

## Results and Discussion

### Catalyst impregnation

**Figure 1:**
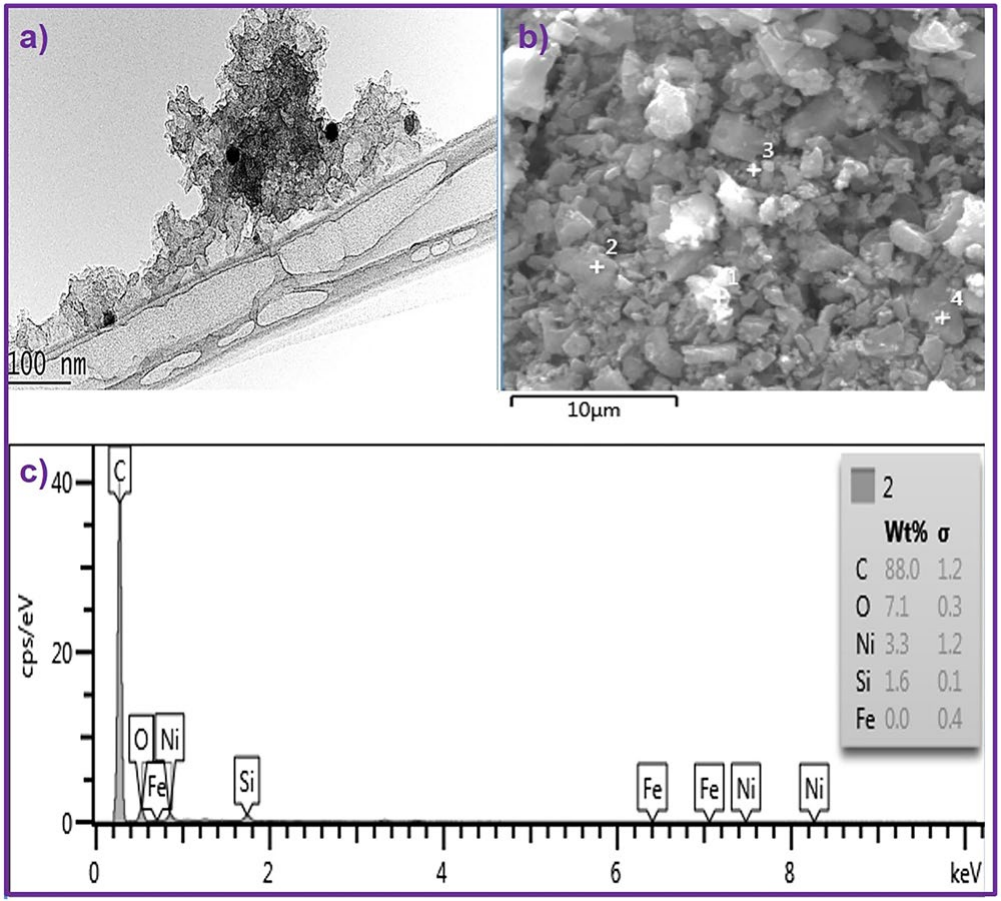
(**a**) TEM image and (**b**) SEM image of Ni doped PAC and (**c**) EDX profile of the selected area of (**b**).

The transmission electron microscope (TEM) image in (Fig. 1(a)) clearly shows that the catalyst was successfully impregnated in the PAC. To further investigate, we performed energy dispersive X-ray analysis (EDX), shown in (Fig. 1(c)), which confirmed that the PAC is comprised of carbon (88 wt%), oxygen (7.1 wt%), Ni (3.3 wt%), and silicon (1.6 wt%). An extremely small amount of Ni was found to be sufficient to facilitate the formation of catalytic sites and to yield CS-free CNFs and CS-mixed CNFs depending on the reaction temperature.

### Model establishment and analysis

The effects of reaction temperature, reaction time, and gas ratio on C_2_H_2_ decomposition were studied using a response surface method (RSM) central composite design (CCD)^31^. A total of 11 runs were performed, and multiple variable analysis i.e. analysis of variance (ANOVA) was used to study the effects on CY and CA. The responses from the resulting 11 runs are shown in Table 1. Using design of experiment (DoE), we fitted the response data of CY and CA to mean, linear, two-factor interaction (2FI), quadratic and cubic polynomial models, as shown in Tables S1 and S2, respectively (Supplementary Information). It was observed that although the coefficient of determination (R^2^) of the linear (0.9293) and 2FI (0.9325) models showed close correlation, the 2FI model was more significant, as its probability, Prob > F, value was calculated as 0.0239 < 0.05^32^. The 2FI model was chosen for CA analysis due to a higher R^2^ (0.9839), although Prob value of mean < 0.05.

**Table 1:** Experimental design matrix and the value of responses based on experiment run.

Run	Temperature °C	Time (min)	Gas Ratio (%)	CY	CA
1	650.00	40.00	2.50	147.7	167
2	550.00	20.00	1.00	159.1	123
3	550.00	60.00	4.00	102.3	112
4	750.00	60.00	1.00	270.6	177
5	650.00	20.00	1.00	91.9	155
6	550.00	20.00	4.00	159.8	97
7	750.00	20.00	4.00	151.9	168
8	750.00	20.00	1.00	92.9	158
9	650.00	60.00	1.00	265.8	173
10	750.00	60.00	4.00	142.2	165
11	550.00	60.00	1.00	196.3	130

### Statistical analysis and modeling

The ANOVA results for the CY responses are displayed in Table 2. As revealed in Table 2, the main effects of the reaction temperature (A), the interaction of the reaction temperature and the gas ratio (AC), and the second-order effects of reaction time (B^2^) were significant, as their Prob > F values were less than 0.05. Therefore, we can conclude that A, AC and B^2^ were the major determinants for CY. The remaining reaction parameters, including the reaction time (B), the gas ratio (C), the interaction of A and B (AB) and the second-order effects of A (A^2^) were shown to have Prob > F values greater than 0.05. Hence, it can be concluded that these parameters had less effect on CY over the studied range. However, these model terms were not eliminated from the analysis in order to ensure that the selected 2FI model remained hierarchical. The regression equation for CY is given by eq. (1).

1 $$\begin{array}{c}CY( \% )=+121.73-6.79+24.29B-9.40C+9.64AB\\ \quad \quad \quad \quad +\,12.61AC-21.11BC+11.68{A}^{2}-23.40{A}^{2}{\rm{B}}\end{array}$$

where *A* represents the reaction temperature, *B* the reaction time, and *C* is the gas ratio.

**Table 2:** ANOVA for CY surface modified model.

Source	Sum of square	DF	Mean square	F	Prob > F
A	7.678E-004	1	7.678E-004	9.20	0.0250
B	0.083	1	0.083	0.070	0.8045
C	0.046	1	0.046	7.59	0.0511
AB	0.086	1	0.086	4.22	0.1093
AC	8.995E-003	1	8.995E-003	7.85	0.0487
A^2^	0.30	1	0.30	0.82	0.4167
B^2^	0.044	4	0.011	27.19	0.0065

**Figure 2:**
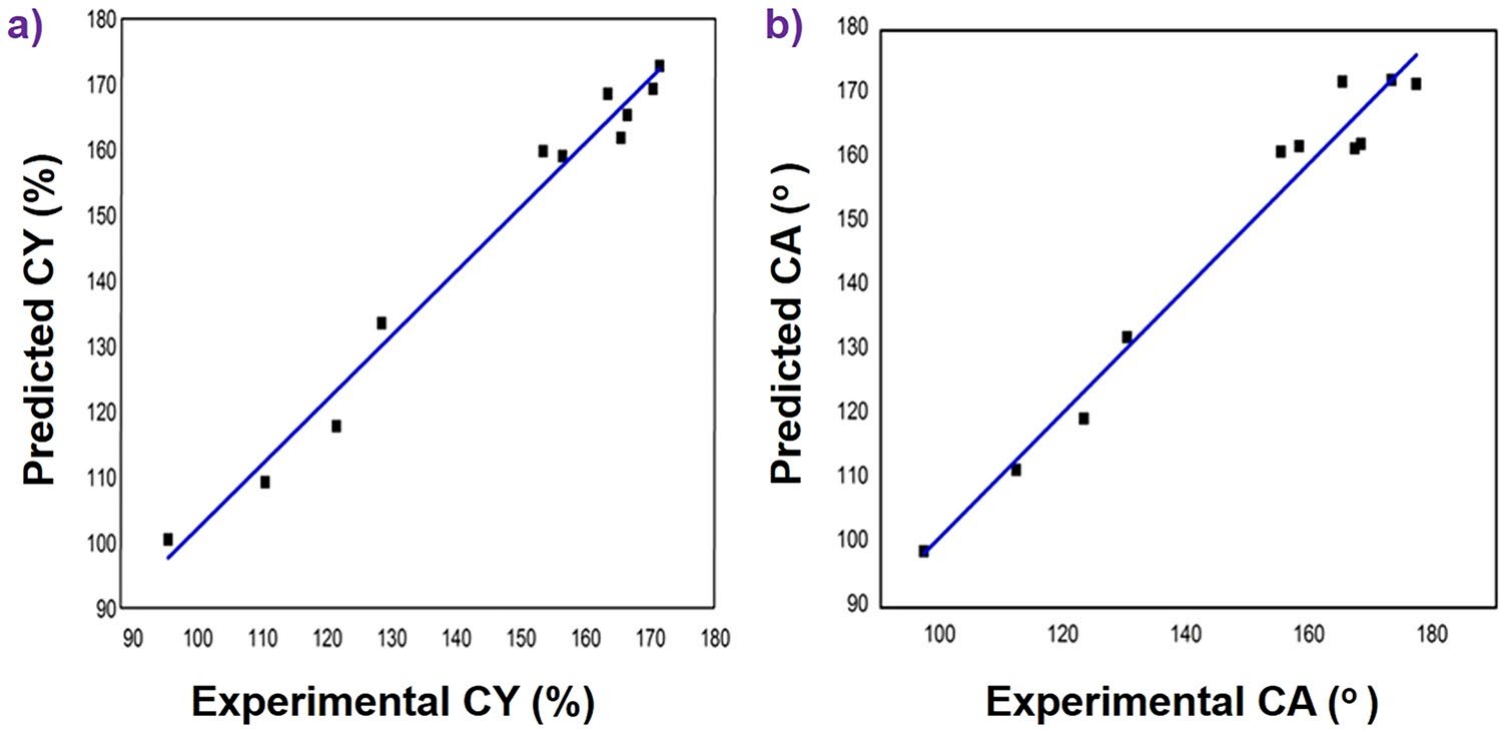
Parity plot of experimental and predicted values of CY (**a**) and CA (**b**).

A comparison between the experimental results and the model values of CY predicted from the above eq. (1) is depicted in (Fig. 2(a)). A simulated model should be congruent with the results, which is indicated by a high R^2^ value. (Figure 2(a)) demonstrates the good convergence between the experimental and predicted values of CY.

The ANOVA results for the CA responses are displayed in Table 3. Similar to CY, the effect of A was found to be significant, with a Prob > F value less than 0.05. Compared to CY, we found significant second-order effects for the reaction temperature (A^2^). Therefore, we can conclude that A and A^2^ were the major determinants of CA. The remaining reaction parameters, such as B, C, the interaction of A and B (AB), the interaction of A and C (AC), and the second-order effect of B (B^2^), demonstrated Prob > F values greater than 0.05. Hence, it can be concluded that these parameters had less effect on CA over the studied range. The regression equation for CA is given by eq. (2).

2 $$\begin{array}{c}CA=+161.62+25.75A+5.60B-5.08C\\ \quad \quad -\,0.75AB+5.25AC-20.37{A}^{2}\end{array}$$

where *A* represents the reaction temperature, *B* the reaction time, and *C* is the gas ratio.

**Table 3:** ANOVA for CA surface modified model.

Source	Sum of square	DF	Mean square	F	Prob. > F
A	5000.00	1	5000.00	125.00	0.0015
B	250.00	1	250.00	6.25	0.0877
C	200.00	1	200.00	5.00	0.1114
AB	18.00	1	18.00	0.45	0.5504
AC	288.00	1	288.00	7.20	0.0748
A^2^	456.33	1	456.33	11.41	0.0432
B^2^	39.38	1	39.38	0.98	0.3942

A comparison of the experimental results with the model values of CA predicted from the above equation is shown in (Fig. 2(b)). A simulated model should be congruent with the results, which is indicated by a high R^2^ value. (Figure 2(b)) demonstrates the excellent convergence between the experimental and predicted values of CA.

### Effects of reaction temperature, reaction time, and gas ratio

**Figure 3:**
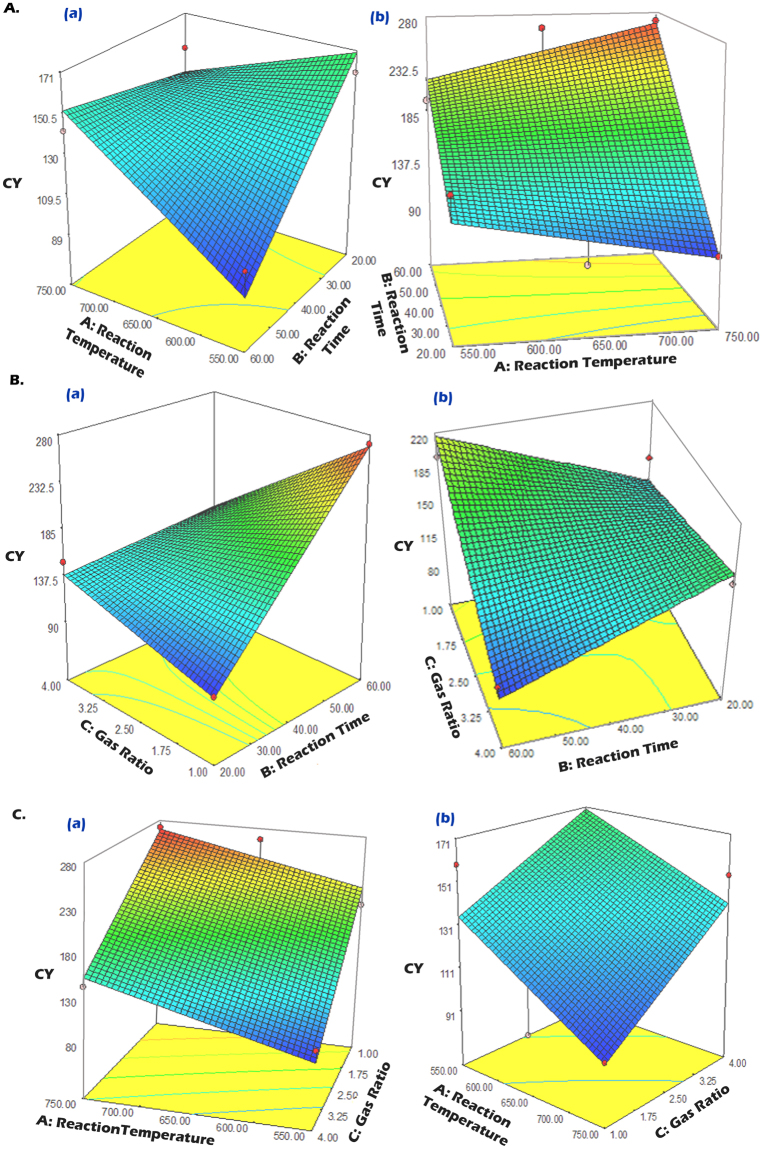
Response surface plots for the effects of reaction temperature and reaction time on CY at fixed gas ratio 4.0 (**Aa**) and 1.0 (**Ab**); effects of reaction time and gas ratio on CY at fixed reaction temperature 750 (**Ba**) and 550 °C (**Bb**); and effects of gas ratio and reaction temperature on the CY at fixed reaction time 60 (**Ca**) and 20 min (**Cb**).

The effects of the process parameters on CY and CA were scrutinized to determine the conditions that favored the desired reaction. The interaction effects of the process parameters on CY and CA were graphically illustrated by CCD curves. These curves were plotted on the basis of the empirical model and are shown for CY in (Fig. 3(A–C)). Figure 3 shows the effects of the reaction temperature and reaction time on CY at fixed gas ratio 4.0 (Aa) and 1.0 (Ab). There was a notable increase in CY with increasing reaction temperatures, from 550 to 750 °C, as shown in both plots. This suggests that conducting the reaction at higher temperatures is recommended, which is in contrast to the results of other studies^32–34^. The Ni catalyst was not deactivated at higher temperatures. This suggests that PAC is an effective support for the Ni catalyst for C_2_H_2_ decomposition even at higher temperatures. Second, the decrease in the growth rate observed in previous studies at lower temperatures was due to progressive blocking of the active surface by excess carbon deposition on the front face of the catalyst particle, which is called catalyst poisoning^35^. This effect can be minimized by creating a PAC network around the Ni catalyst. Figure 3 shows the effects of the reaction time and gas ratio on CY at fixed reaction temperature 750 (Ba) and 550 °C (Bb). There was a steady increase in CY with increasing reaction time for a gas ratio of 4.0 at high temperature (Fig. 3(Ba)). Typically, the rate of diffusion for carbon into the catalyst decreases with increasing reaction time and eventually approaches zero, giving rise to no additional increase in the length or the yield^35^. The increased CY in this study may be due to an increase in the rate of C_2_H_2_ decomposition because the activity of the Ni catalyst remained high due to higher diffusivity of carbon atoms on the PAC-protected surface of the Ni catalyst particles exposed to a gaseous environment. However, a high reaction time was unfavorable at the maximum gas ratio of 4.0 at low temperature (Fig. 3(Bb)). Figure 3 shows the effects of the reaction temperature and gas ratio on CY at fixed reaction time 60 (Ca) and 20 min (Cb). Here, it can be seen that the effect of the gas ratio on CY was not significant, as a higher CY was seen at the gas ratio of 1.0 at high temperatures. This indicates that the gas ratio does not influence nucleation for carbon growth, while PAC has been used as a support for Ni catalysts at high temperatures for long reaction time, i.e. 60 min (Fig. 3(Ca)). It is well known that the presence of a higher H_2_ partial pressure, along with C_2_H_2_, can suppress catalyst poisoning effects, which in turn results in an increase in the length of CNM^36^. H_2_ has been recognized an ideal energy carrier in CVD processes and correlates with the structure of CNM. Any carbon atom that diffuses into the cluster will be diluted, which affects the amount of CNM that are formed. H_2_ gas has been shown to help control the deposition quality of carbon soot and, ultimately, the quantity of CNM. However, our study also indicates that the suppression of catalyst poisoning could be potentially minimized by using PAC, which would protect the Ni catalyst even at a higher flow of H_2_/C_2_H_2_ gas.

**Figure 4:**
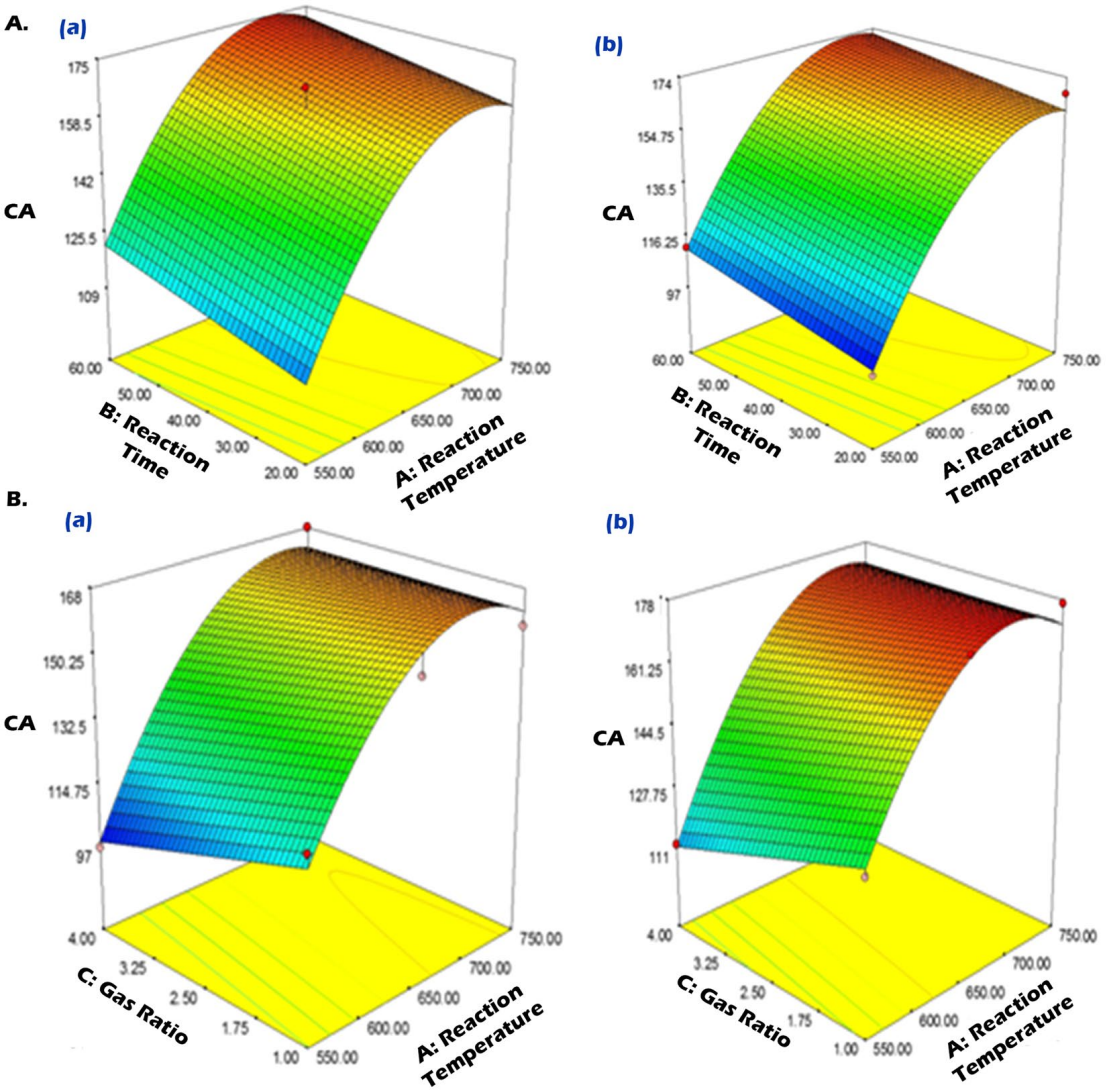
Response surface plots for the effects of reaction temperature and reaction time on CA at fixed gas ratio 4.0 (**Aa**) and 1 (**Ab**); and effects of reaction temperature and gas ratio on CA at fixed reaction time 60 (**Ba**) and 20 min (**Bb**).

Figure 4 shows the effects of the reaction temperature and reaction time on CA at fixed gas ratio 4.0 (Aa) and 1.0 (Ab). Similar to CY, there was a notable increase in CA with increasing reaction temperatures, from 550 to 750 °C, as shown in both plots. The reaction time also showed similar effects. Figure 4 presents the effects of the reaction temperature and gas ratio on CA at fixed reaction time 60 min (Ca) and 20 min (Cb). It can be observed that the effects of the gas ratio on CA were negligible, as the lowest CA was seen at the gas ratio of 4.0 at high temperature, as shown in both plots. Note that an increased gas ratio can increase CY only a low reaction time 20 min (Fig. 3(Cb)) due to the generation of longer CNF production^36^. Alternatively, increasing the gas ratio can decrease CA may be due to the stimulation of CNF production, rather than CS. We found that extreme hydrophobicity largely depends on CS production in the CNF network, as it increases the surface roughness. This is well corroborated by the TEM and CA analyses.

### Optimization study

The independent factors and responses considered important in C_2_H_2_ decomposition were optimized simultaneously using a numerical optimization method in the DoE software. All the factors and responses corresponding to the upper and lower limits of the experimental range had to satisfy the criteria defined for the optimum working conditions. The ultimate goal for C_2_H_2_ decomposition into CNM is to maximize the CY and CA. Several sets of combinations were predicted to be possible optimized conditions, which were further ranked by desirability. The optimum process conditions for the highest desirability were a reaction temperature of 702 °C, a reaction time of 40 min, and a gas ratio of 1.0, which gave the highest CY of 380%. Similarly, a reaction temperature of 687 °C, a reaction time of 40 min, and a gas ratio of 1, gave the highest CA of 177°. The experiments were performed at the predicted optimum conditions to experimentally verify the CY and CA results. These results show that the predicted CY and CA were close to the experimental values, with a mean error of 2.21%.

### FE-SEM and TEM analyses

**Figure 5:**
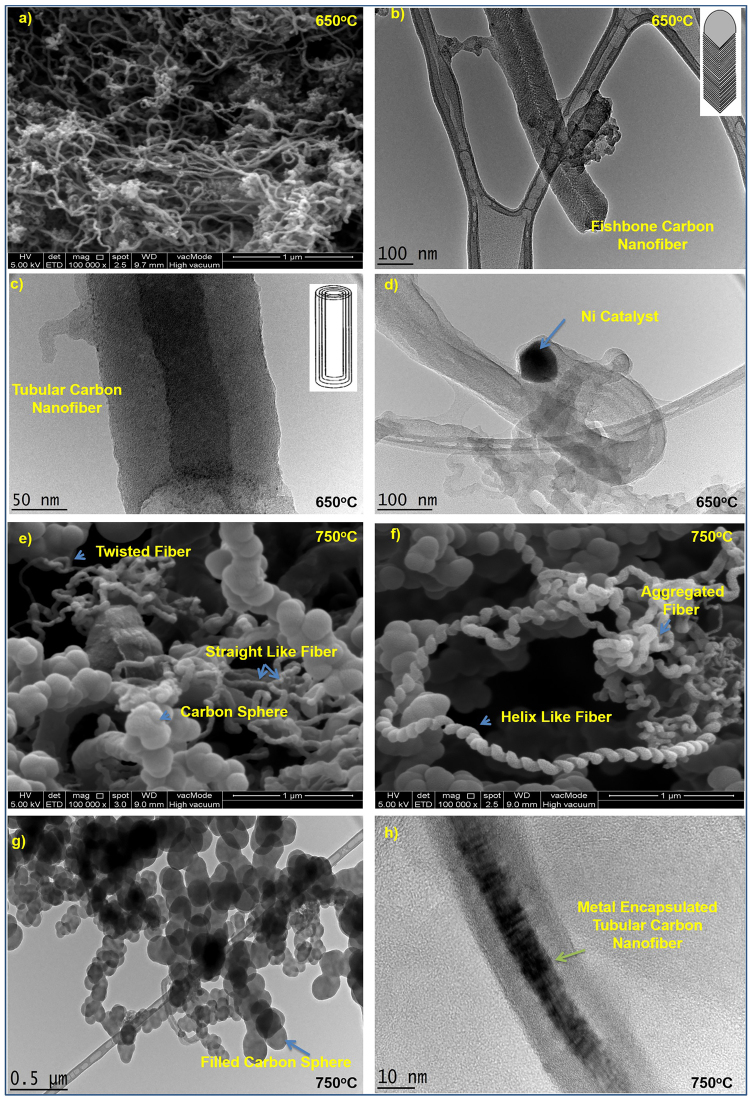
FE-SEM image of CNF (**a**) cum TEM images of CNF (**b**–**d**) obtained at 650 °C; and FE-SEM images of CNF, helix and CS (**e**,**f**) cum TEM images of CS (**g**) and of metal encapsulated CNF (**h**) produced at 750 °C.

The morphologies of the deposited products, obtained using PAC/Ni, are shown in (Fig. 5). At 650 °C, only CNF-like materials were observed, as shown in the Field emission scanning electron microscope (FE-SEM) image (Fig. 5(a)). However, the TEM images suggested that two types of CNF were produced, specifically fishbone-type CNFs (Fig. 5(b)) and tubular CNFs (Fig. 5(c))^37^. Interestingly, no single predominant type of CNF was observed at 750 °C, as shown in the FE-SEM images (Fig. 5(e,f)) and TEM images (Fig. 5(g,h)). We observed fibers in the forms of ribbons, which were possibly twisted into a helix (Fig. 5(f)), and metal-encapsulated tubular CNFs (Fig. 5(h)). In addition, we observed a considerable amount of CS (Fig. 5(e–g))^37^. The average CNF diameter was shown to increase from 20–30 nm at 650 °C, as shown in (Fig. S1), to 40–55 nm at 750 °C, as shown in (Fig. S2(a)). This may be due to sintering of the Ni catalyst, facilitated by the decomposition of PAC functional groups^38^. The predominant CNF helix diameter was 130–170 nm at 750 °C, as shown in (Fig. S2(b)). In addition, CNFs synthesized at both 650 and 750 °C were found to consist of graphitic layers as confirmed from (Fig. 5(c,h)). It should be noted that previous studies have claimed that only one type of CNF was found when PAC was used as the substrate for metal catalysts^18–21^. This is possibly due to the use of C_2_H_4_ rather than C_2_H_2_ as well as the different reaction temperatures. As shown in (Fig. 5(d)), CNF precipitated across the Ni bottom and pushed the entire Ni particle off the PAC, suggesting a “base or root” growth model. This occurred because of the weak interaction between Ni and PAC, since the Ni catalyst was physically adsorbed on PAC. Therefore, the overall CNF growth process can be considered to occur in three main steps as shown in (Fig. S3): i) the carbon feedstock was supplied to the Ni/PAC surface to obtain an intermediate; ii) small carbon fragments, such as C_2_ and C_3_, were generated from the decomposition of C_2_H_2_ at 650/750 °C and subsequently deposited on the surface of the Ni catalyst; and iii) CNFs grew until the Ni was fully covered with excess carbon, where after the catalytic activity ceased and CNF growth stopped.

In addition to CNF, the synthesis of CS at 750 °C is another advantage of this study. Recently, there has been renewed interest in the synthesis and study of CS, with many scientists now attempting to prepare CS due to their high surface area, thermal stability, unique electronic properties, low density, and most importantly, their tailored structure^39^. CS have been used in lithium batteries, as catalyst supports, in drug delivery, for the encapsulation of active transition metals, and as lubricants^39,40^, to name a few. As shown in (Fig. 5(e,g)), CS differs from fullerene in that they are solid. Since the diameter of the obtained CS fell between 80 and 130 nm (Fig. S2(c)), one can classify these as less graphitized spheres (50–1000 nm)^39^. As shown in (Fig. 5(e,g)), all the CS tend to be connected, forming bead- or necklace-like structures, through van der Waals forces, which leads to agglomerated collections of CS. A possible growth mechanism of CS formation is shown in (Fig. S4). A detailed mechanism of carbon nucleation to produce CS was outlined by Deshmukh *et al*.^39^.

### Raman analysis

**Figure 6:**
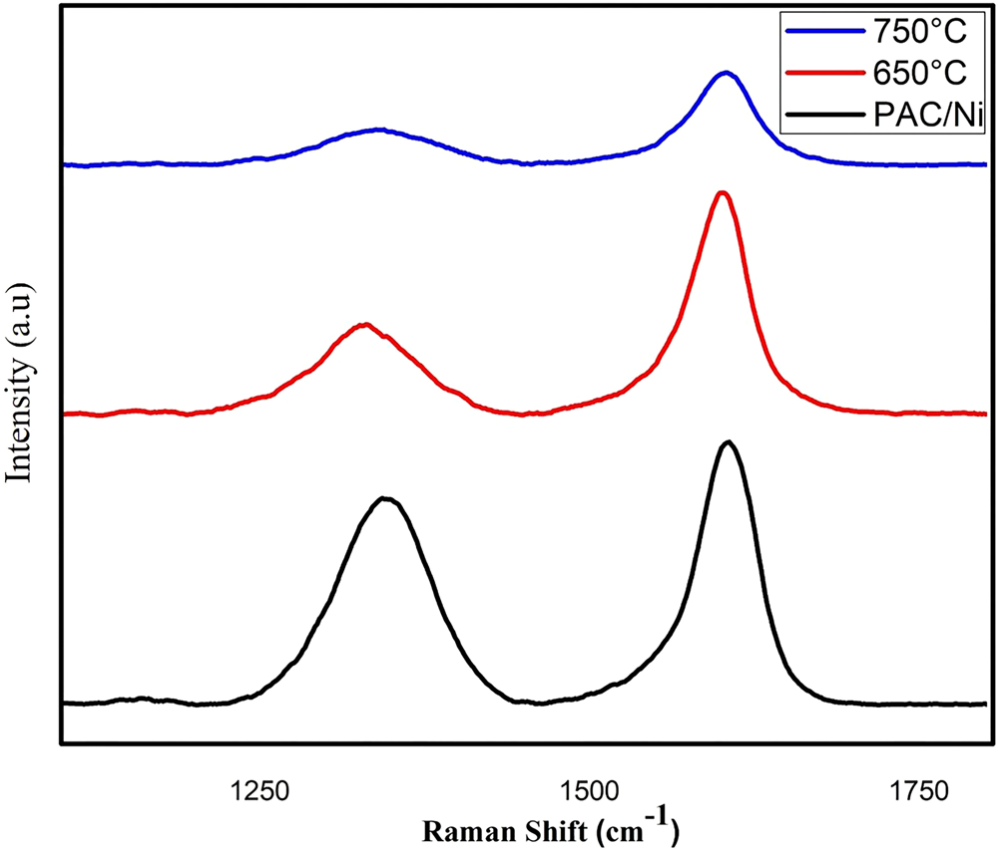
Raman spectra of PAC/Ni and CNM produced at 650 and 750 °C.

We used Raman spectroscopy to characterize the nature of the present carbon. The peak at around 1593 cm^−1^ (G band) corresponds to the E2g mode of hexagonal graphite and was observed for all CNM as shown in (Fig. 6). This is related to the vibration of sp^2^-hybridized carbon atoms in a graphite layer^41^. This suggests that, similar to the CNFs, the CS were also composed of graphitic carbon, which is consistent with the previously discussed TEM and FE-SEM results. In addition, the band in the obtained CNM spectra at around 1314 cm^−1^ (D band) suggests the presence of defective amorphous carbon structures^42,43^. The relative intensity of the G and D bands can be related to the organizational degree of the carbon material. Ni doping did not change the overall structure of PAC, since it shows an ID/IG of 0.81. The IG/ID ratio observed for the CS-free CNFs at 650 °C (0.74) is slightly larger than that for the CS-mixed CNFs at 750 °C (0.70). It suggests the CS-free CNF consists of well-graphitic structure than that of CS-mixed CNF^44^.

### FTIR analysis

**Figure 7:**
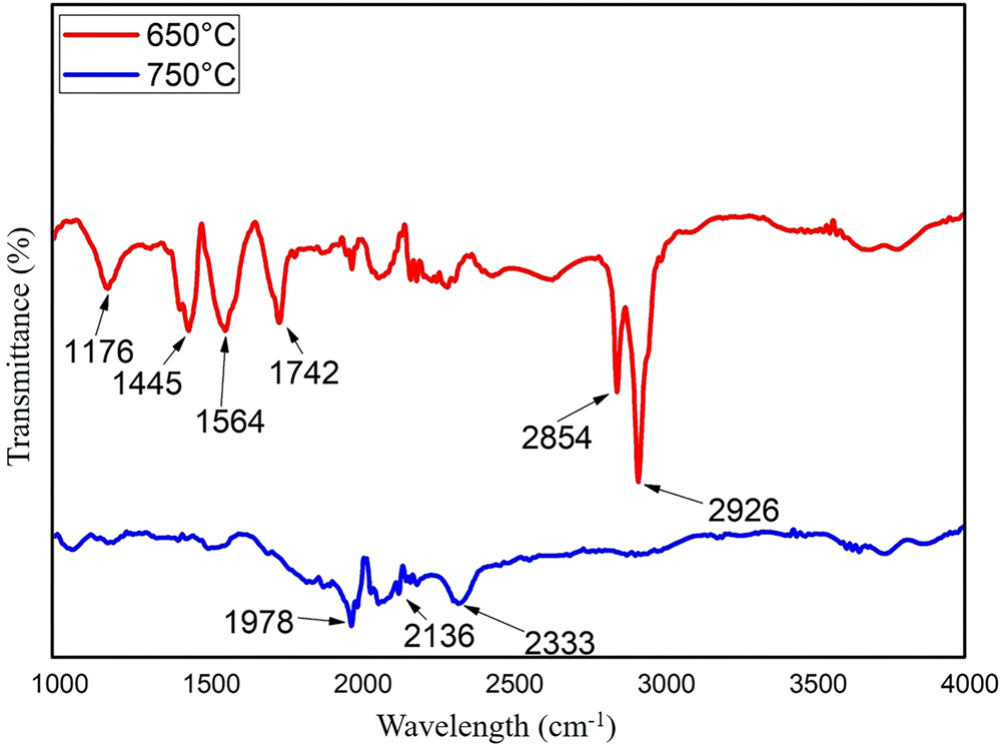
FTIR spectroscopy of CNM produced at 650 and 750 °C.

Fourier transform infrared (FTIR) spectroscopy was used to investigate the surface chemistry and functional group quality of the CNM as shown in (Fig. 7). The FTIR spectrum of the CS-mixed CNFs (750 °C) illustrates only a few functional groups. Peaks at 1978 and 2136 cm^−1^ might be attributed from metal (Ni) carbonyl, whereas characteristic peak at 2333 cm^−1^ was due to adsorbed CO_2_^45^. IR spectrum of CS-free CNF (650 °C) showed a range of functional groups. For example, peak at 1176 cm^−1^ was due to CO (epoxy or alkoxy). The dominant peaks at 1445 and 1564 cm^−1^ were appeared due to stretching vibrations of CNF aromatic rings^45^ and C=C stretch^46^, respectively. The emerging peaks at wave numbers 1742 cm ^−1^ was associated with C=O group^46^. Relatively weak and strong peaks at 2854 and 2926 cm ^−1^ were may be due to –CH and CH_2_ stretching^47^. All of these high-intense peaks were absent in CS-mixed CNF (750 °C) which suggests the functional groups were only sponsored by CNF rather than CS. In general, CNF is contaminated with amorphous carbon during the growth process in CVD which typically oxidized by air to produce a few functional groups^48^. Such observation is in agreement with the results obtained by the measurement of the zeta-potential of the CS-free CNFs (650 °C), as we discuss in “CA and hydrophobicity analyses” section. Typically, oxy-functional groups led to more negative values, and as seen in the CA measurement results, the CS-mixed CNFs (750 °C), which has a few of functional groups, should demonstrate higher hydrophobicity.

### TGA

**Figure 8:**
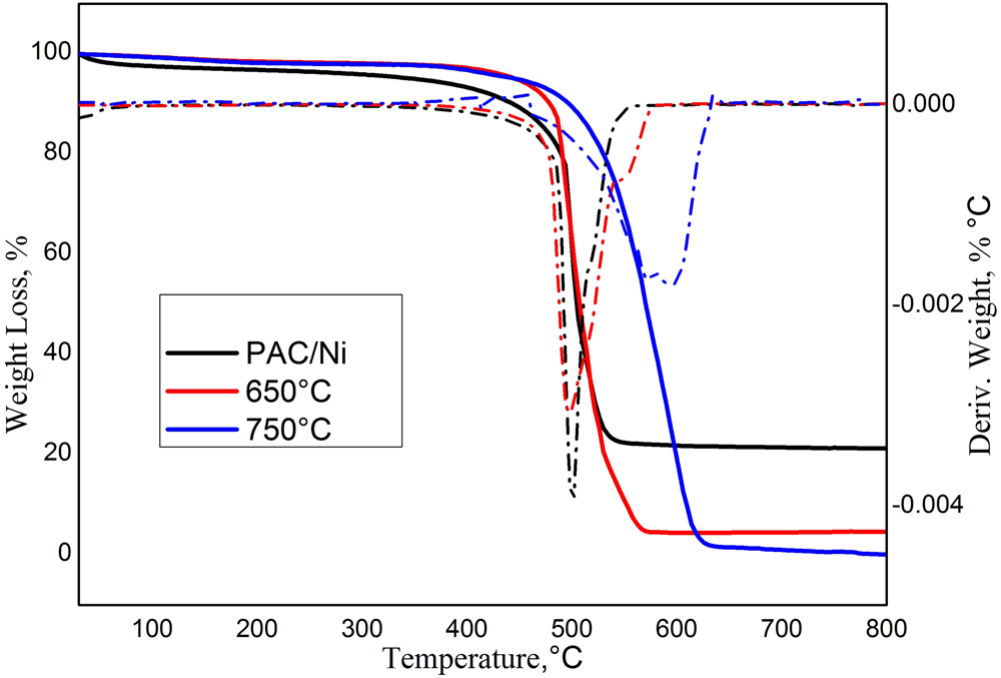
TGA and DTG spectra of PAC/Ni and CNM produced at 650 and 750 °C.

Thermogravimetric analysis (TGA) was performed to measure the purity, defects, and overall quality of the obtained CNM. The TGA of the produced CNM with their derivative spectra are presented in (Fig. 8). The oxidation temperature represents the temperature at which CNM loses weight and shows the highest derivative weight curve. This reflects the stability of CNM at a given temperature. No weight loss was observed for PAC/Ni or the CNM at 650 or 750 °C at degassing temperatures ranging from 100 to 400 °C. PAC/Ni degradation began at approximately 500 °C and resulted in a loss of nearly 80 wt%. Typically, amorphous carbon oxidizes at approximately 500 °C^49^ due to its lower activation energy and the presence of numerous heat-sensitive active sites^50^. The presence of oxy-functional groups in the CS-free CNFs (650 °C) was confirmed by FTIR spectroscopy (Fig. 7) and may be responsible for the weight loss at low decomposition temperatures, i.e., just slightly over 500 °C, compared to the CS-mixed CNFs (750 °C), which showed 100 wt% loss at just above 600 °C^51^. The ash content of the CS after combustion above 600 °C was 0 wt%, implying that the produced CS was less graphitized in structure^41^.

### CA and hydrophobicity analyses

**Figure 9:**
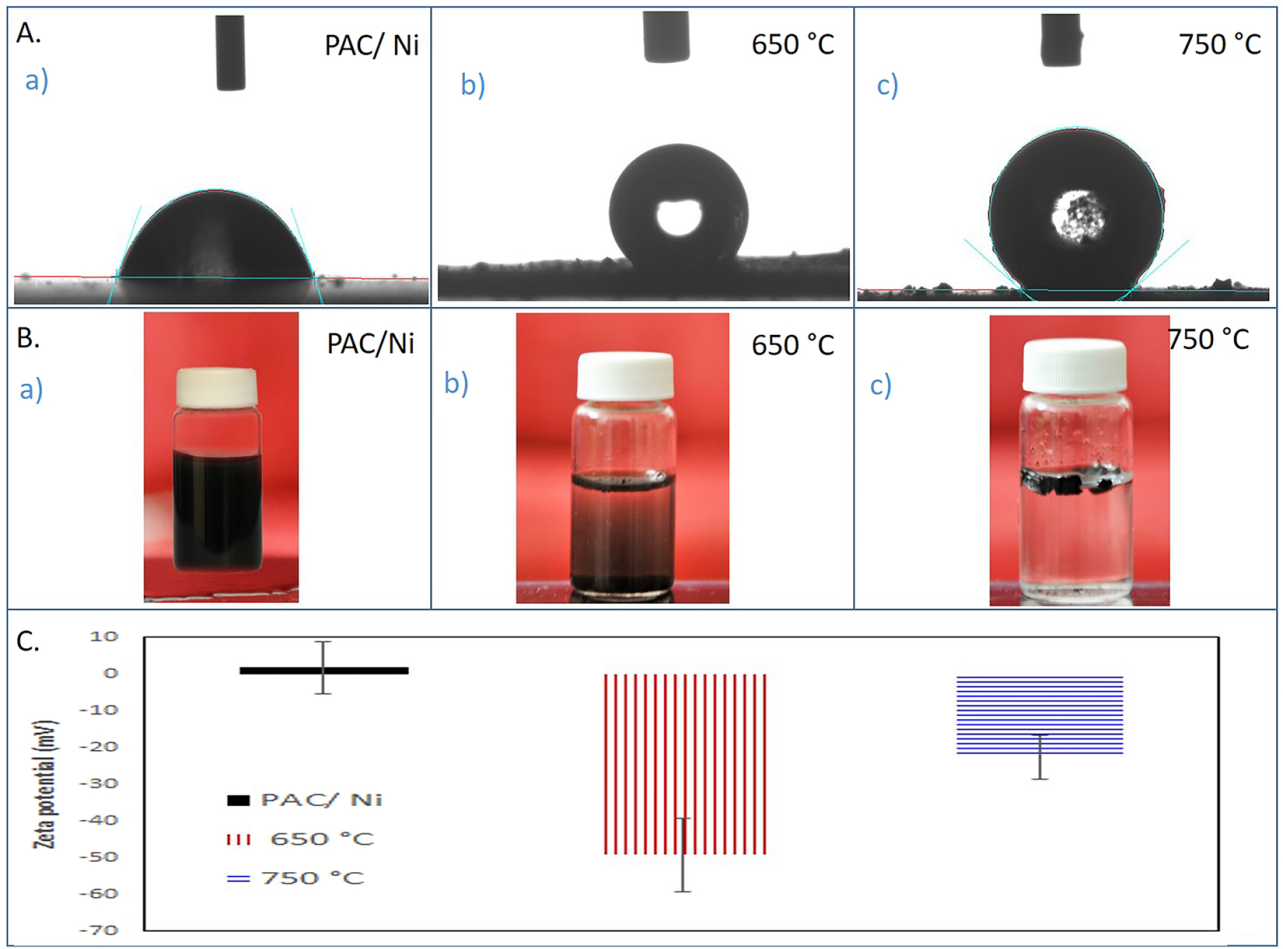
CA (**A**) for the (a) PAC/Ni (65°), (b) CS free CNF (167°) and (c) CS mixed CNF (177°). Colloidal stabilities (**B**) of (a) PAC/Ni, (b) CS-free CNT at 650 °C and (c) CS-mixed CNF at 750 °C. Zeta potential measurements of PAC/Ni, CS-free CNT at 650 °C and CS-mixed CNF at 750 °C.

Superhydrophobic surfaces with a water CA higher than 150° have drawn great scientific and industrial interest^7^. We studied the hydrophobicity of PAC/Ni, CS-free CNF and CS-mixed CNF as shown in (Fig. 9(A)) (measured within 1 min of placing the drop on the sample). Since PAC/Ni is not a hydrophobic material, the PAC/Ni solution cast film shows a CA of 65° (Fig. 9(A(a)), and the lower apparent CA (167°) of the CS-free CNFs (650 °C) (Fig. 9(A(b)) is not much more hydrophobic than the CA (177) of CS-mixed CNFs (750 °C) (Fig. 9(A(c)). This suggests that the formation of CS could be a possible cause of the high hydrophobicity of the CS-mixed CNF surface, as the droplet rests on the heterogeneous CNF surface. The surface wettability of CNM can be thoroughly explained by two different factors, specifically the surface chemistry and the surface roughness^10^. First, since the CS-free CNFs (650 °C) contain oxygenated functional groups, as confirmed by FTIR (Fig. 7), they are capable of attracting water molecules on their surface, compared to the CS-mixed CNFs (750 °C), which contain a few functional groups. Second, superhydrophobic surfaces require a certain surface roughness, the effect of which is seen in the apparent contact angle, θ°^52^. The growth of CS on the CNF network imparts a rougher surface area compared with that of the CS-free CNFs and minimizes the cavities available to water droplets. These explanations have also been experimentally proven by Ma *et al*., who suggested that high-density CS are more hydrophobic than CS-free CNFs^9^.

Optical observations of the colloidal stability of the CS-free CNFs and CS-mixed CNFs are shown in (Fig. 9(B)). The CS-free CNFs were not well dissolved in neutral water and were found to precipitate rapidly to the bottom of the vial (Fig. 9(B(b)). However, the CS-mixed CNFs showed extreme hydrophobicity, and the material even floated on the water surface (Fig. 9(B(c)). These results agreed with the CA and ζ-potential analyses. The ζ-potential is the electric potential at the interfacial double layer, which is important in measuring the surface charge of the material, as shown in (Fig. 9(C)). The values between 0 and −49 obtained for the CS-free CNFs (650 °C) indicated the mutual repulsion of CNFs, which is due to the high surface charge, providing considerable solubility^53^. On the other hand, the ζ-potential values between 0 and −20 mV indicate the onset of the agglomeration of the CS-mixed CNFs and their floatability. The hydrophobic surfaces of CS-mixed CNFs do not favor bond formation with water molecules, and thus, they do not disperse in water (Fig. 9(B(c)).

## Conclusion

RSM showed the interactions of different parameters in the process of CNM growth on PAC by CVD. CY and CA were taken as the responses, and a significant regression equation was obtained, which led to a successful optimization. Based on the results from the experimental design, we conclude that temperature plays an important role and that the reaction time was significant factors for CY. Meanwhile, the main experimental factor for CA was the reaction temperature. The optimization indicated that the best conditions were a reaction temperature of 702 °C, a gas ratio of 1, and a reaction time of 40 min, which gave the highest CY of 380%. Similarly, a reaction temperature of 687 °C, a gas ratio of 1, and a reaction time of 40 min led to the highest CA of 177°. As revealed by the SEM and TEM analyses, the CNM produced under optimal conditions produced CS-free CNF and CS-mixed CNF at 650 and 750 °C, respectively. It was also observed in the TEM and SEM images that CNM were densely grown on the catalyst. In addition, Raman analysis discovered that these CNM possessed a high degree of graphitization and few defects. This study illustrates, through the CA on the CNM surface, that a stable superhydrophobic material was formed with high CY.

## Materials and Methods

### Materials and reagents

Nickel (II) nitrate hexahydrate, PAC, acetone, C_2_H_2_, H_2_, and N_2_ were purchased from Sigma Aldrich, Malaysia. All chemicals were of analytical grade.

### Synthesis of CNM

#### Catalyst impregnation

PAC (2 g) was added to 5 mL of nickel (II) nitrate hexahydrate (1 wt%) acetone solution and sonicated at 40 KHz for 1 h at 50 °C. After achieving a uniform dispersion of PAC, the acetone was evaporated, and the Ni-doped PAC was dried at 100 °C. The dried sample was thoroughly ground, and the resulting powder was then stored in a desiccator. The PAC-Ni samples were calcinated at 350 and 700 °C for 2 and 1 h, respectively, under an inert atmosphere (N_2_, 200 mL/min).

### Growth of CNM using CVD

The CNM-PAC superstructure was synthesized by placing the Ni-doped PAC into a ceramic boat containing CVD reaction tubes^16,18^. The temperature was set within the 550–750 °C range. Then, C_2_H_2_ mixed with H_2_ at a ratio of 1–4 was passed through the reaction tube for 20–60 min. After growth of the CNM, the reactor was cooled under N_2_ flow (200 mL/min), the deposited CNM were collected from the ceramic boat, and the yield was calculated using eq. (3).

3 $$Yield=(Wp-Wc)/Wc$$

where *W*_*P*_ and *W*_*C*_ are the weight of the sample after and before the reaction, respectively.

### Experimental design and optimization of CY and CA

To obtain the optimal CY and CA values, we investigated the growth conditions for CNM using the DoE (CCD Version 7), namely, the reaction temperature, gas ratio, and reaction time. The optimization criteria involved the maximization of CY and CA and the minimization of the time and temperature. Prior to calculating the optimum solution, a statistical regression model was derived through the DoE software, through which a number of runs were eliminated. In addition, ANOVA was performed to determine the significance of the model and interactions of the various parameters.

### Characterizations

The surface morphologies and topologies of the Ni-doped PAC, CS-free CNFs, and CS-mixed CNFs were examined using FE-SEM (Hitachi-SU8000, Japan) and TEM (Hitachi-HT7700, 120 kV, Japan). For these experiments, the CNM were dispersed into fresh MilliQ water and mounted onto lacey copper grids for FE-SEM and TEM analyses. EDX combined with SEM (Quanta FEG 450, USA) was performed to confirm the Ni content of PAC using an X-Max silicon drift detector at 10 KeV. The Raman spectra of the CNM were acquired for 10 min at a laser power of 100 using an Ar + laser (514 nm) focused (50× objective) to a spot size of approximately 1.5–2.0 µm (Renishaw in Via, UK). FTIR spectroscopy, using a PerkinElmer® FTIR spectrometer, was used to study the surface modifications. The measured spectra were collected using an exposure time of 10 seconds with a laser power of 100. TGA was conducted under an O_2_ flow between 25 to 1000 °C at a heating rate of 100 °C/min (TGA/SDTA 851, Mettler Toledo, USA).

### CA and zeta potential measurements

The CA between the CNM and water was calculated by placing a drop of water (4 µL) onto a glass microscope slide (76 × 26 × 1.2 mm) covered with double-sided adhesive tape and measuring the CA with a KRUSS Goniometer (DSA100). Each measurement was repeated in triplicate, and the average was taken. The zeta-potential, or surface charge, analysis of a 0.01 Wt% CNM suspension was determined using a Zetasizer Nano ZS instrument (Malvern, model ZEN3600) (United Kingdom).

## Electronic supplementary material

Supplementary Information
