# Supplementary material for: Optimization of the Synthesis of Superhydrophobic Carbon Nanomaterials by Chemical Vapor Deposition
Source: Sci Rep. 2018 Feb 9;8:2778. doi: 10.1038/s41598-018-21051-3 (PMC5807387; doi:10.1038/s41598-018-21051-3)
Supplement: Supplementary file 1 — Supplementary Information [file 41598_2018_21051_MOESM1_ESM.pdf]

Supplementary File

Optimization of the Synthesis of Superhydrophobic Carbon Nanomaterials by Chemical Vapor Deposition

Mustafa Mohammed Aljumaily[a,b], Mohammed Abdulhakim Alsaadi[a,b,c]*, Rasel Das[a*], Sharifah Bee Abd Hamid[a], N. Awanis Hashim[d], Mohamed Khalid AlOmar[b,e], Haiyam Mohammed Alayan[b,d], Mikhail Novikov[a], Qusay F Alsalhy[f], Mohd Ali Hashim[b,d]

[a]Nanotechnology & Catalysis Research Centre (NANOCAT), IPS Building, University of Malaya, 50603 Kuala Lumpur, Malaysia.

[b]University of Malaya Centre for Ionic Liquids, University Malaya, Kuala Lumpur 50603, Malaysia.

[c]National Chair of Materials Sciences and Metallurgy, University of Nizwa, Sultanate of Oman.

[d]Department of Chemical Engineering, University of Malaya, Kuala Lumpur 50603, Malaysia.

[e]Department of Civil Engineering, University of Malaya, Kuala Lumpur 50603, Malaysia.

[f]Membrane Technology Research Unit, Chemical Engineering Department, University of Technology, Alsinaa Street No. 52, B. O. 35010, Baghdad, Iraq.

*E-mail: mdsd68j@gmail.com, Tel: +60163630693, Fax: +60 3 7967 5311(Alsaadi, M.A.), and raseldas@daad-alumni.de (Das, R.)

Table S1: Sequential model sum of squares for CY						
**Source**	**Sum of squares**	**DF**	**Mean square**	**F value**	**Prob >F**	**R2**
**Mean**	2.882E+005	1	2.882E+005	-	-	0.3860
**Linear**	14557.64	3	4852.55	1.47	0.3036	0.9293
**2FI**	20484.06	3	6828.02	10.24	0.0239	0.9325
**Quadratic**	121.66	2	60.83	0.048	0.9544	0.3860
**Cubic**	2546.03	2	1273.01	-	-	-

Table S2: Sequential model sum of squares for CA						
**Source**	**Sum of squares**	**DF**	**Mean square**	**F value**	**Prob >F**	**R2**
**Mean**	2.401E+005	1	2.401E+005	11.66	0.0041	0.8333
**Linear**	6258.32	3	2086.11	0.37	0.7819	0.8693
**2FI**	270.07	3	90.02	7.13	0.0628	0.9839
**Quadratic**	860.96	2	430.48	-	-	-
**Cubic**	120.83	2	60.42	-	-	-

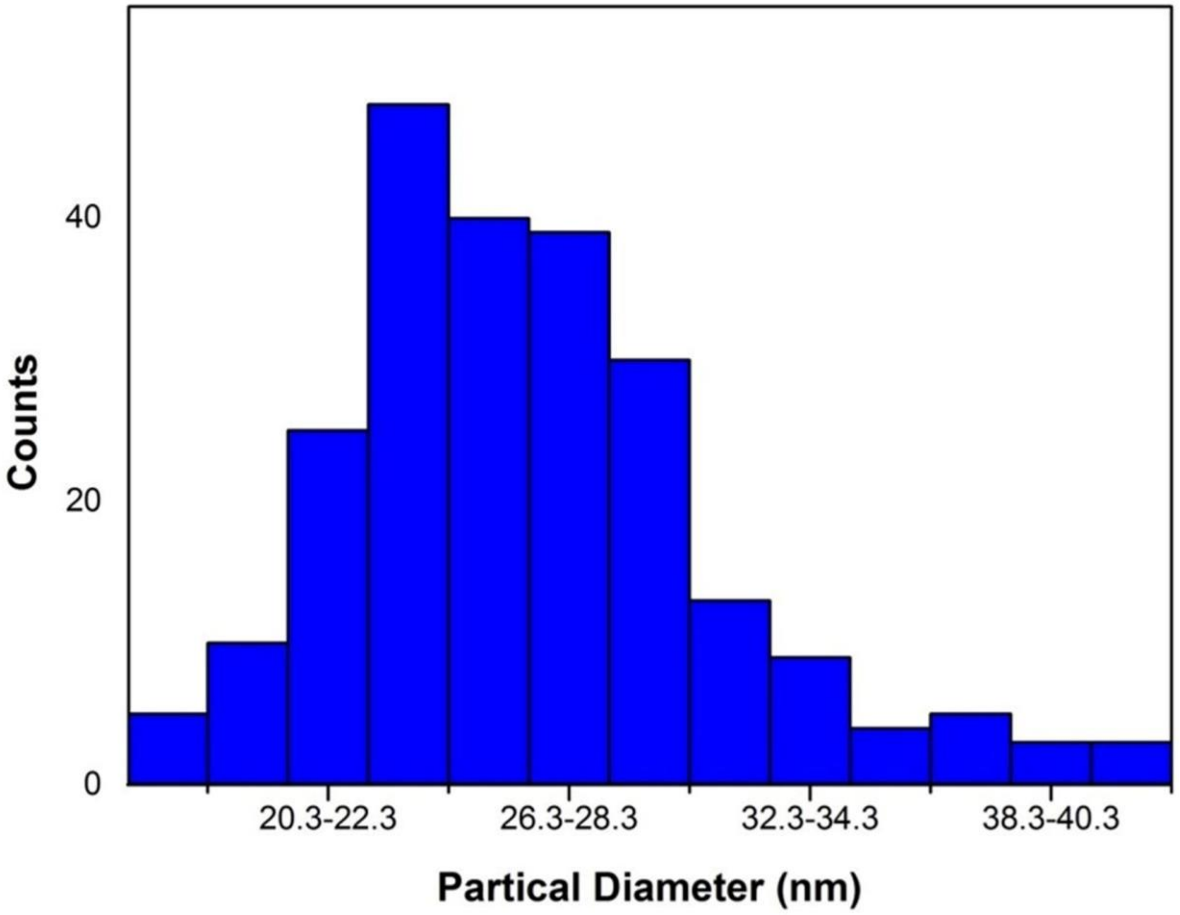

Figure S1: The curve distribution of CNF of 650 °C.

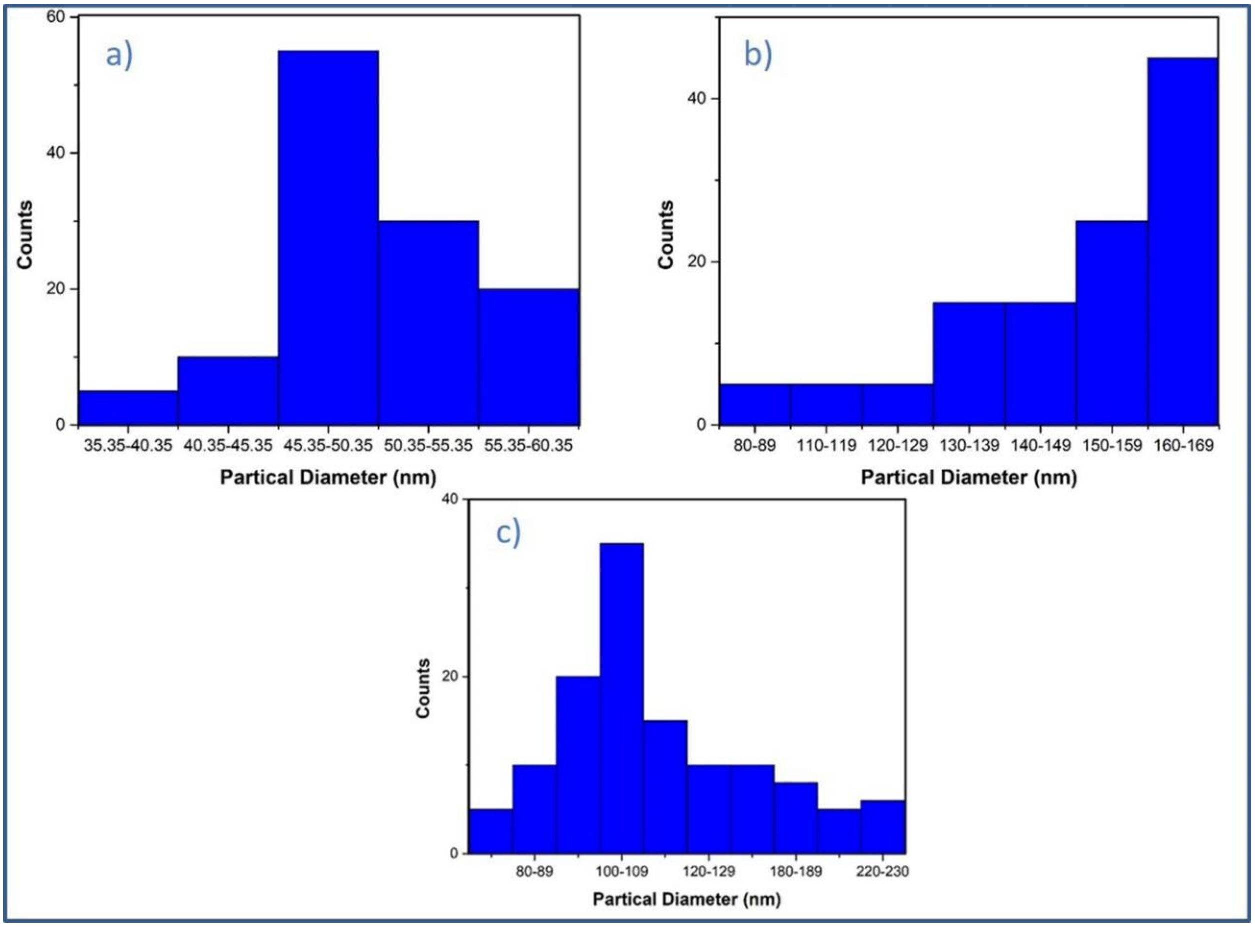

Figure S2: The curve distributions of (a) straight-like CNF, (b) CNF helix and CS (c).

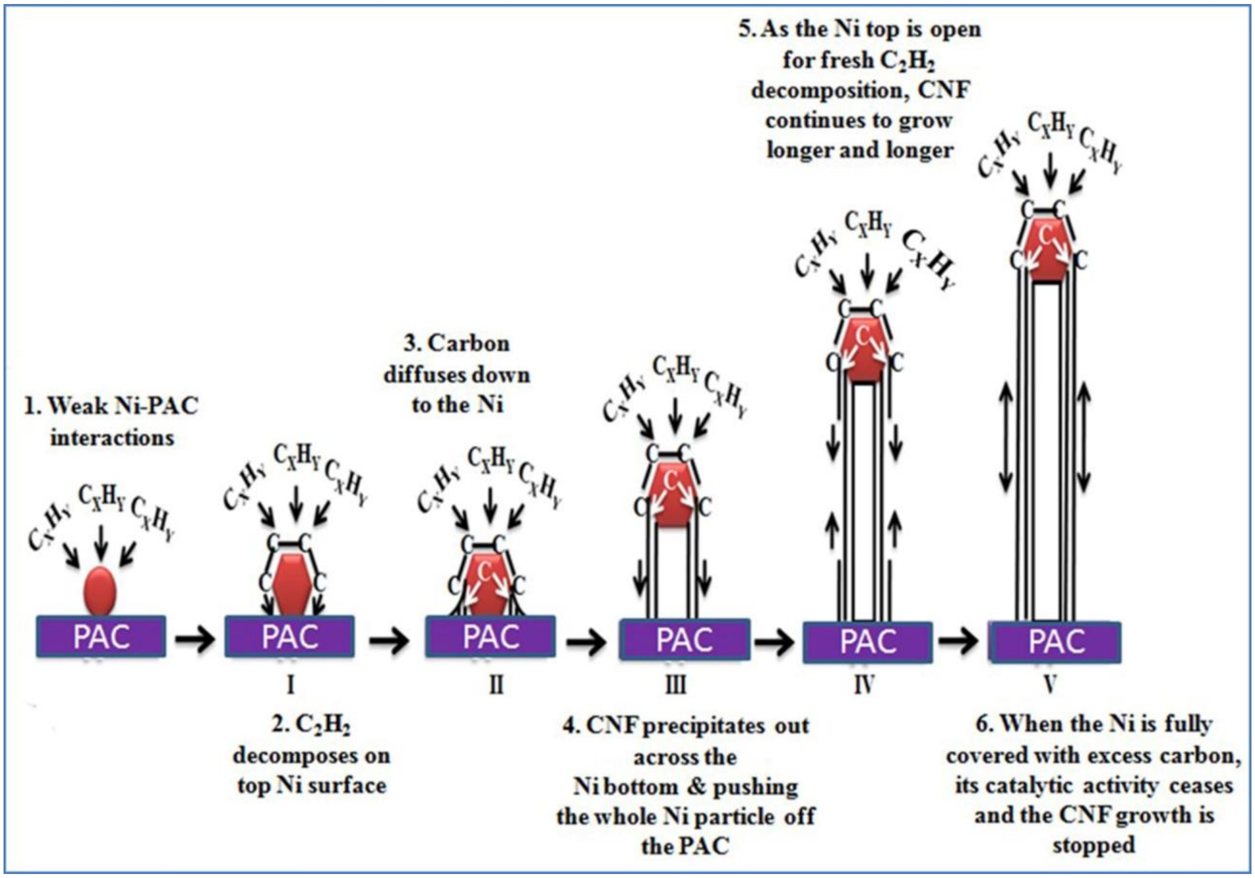

Figure S3: CNF growth mechanism based on CNT nucleation model.

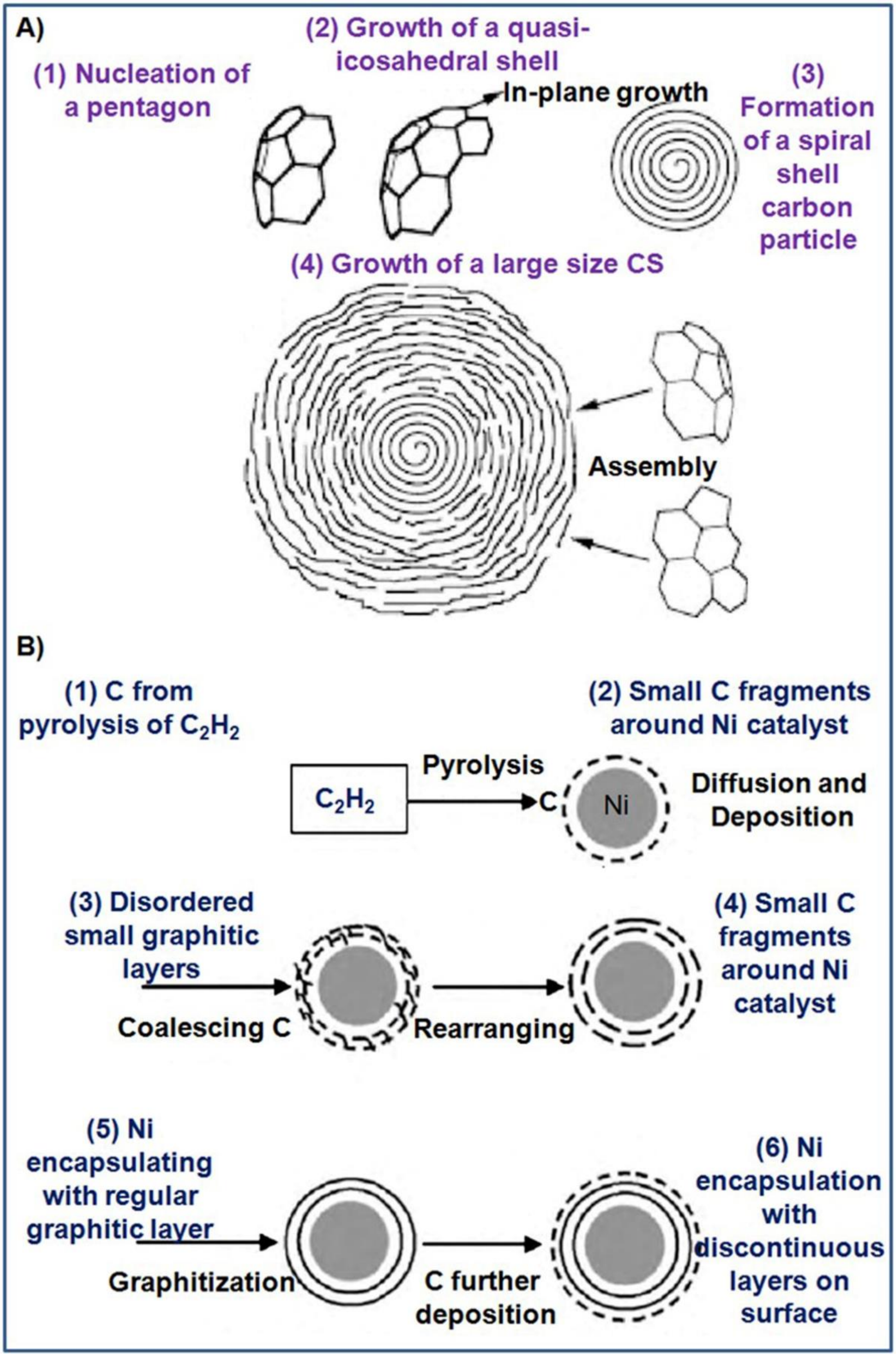
A)
(1) Nucleation of a pentagon
(2) Growth of a quasi-icosahedral shell
In-plane growth
(3) Formation of a spiral shell carbon particle
(4) Growth of a large size CS
Assembly
B)
(1) C from pyrolysis of C_2H_2
(2) Small C fragments around Ni catalyst
C_2H_2
Pyrolysis
C
Ni
Diffusion and Deposition
(3) Disordered small graphitic layers
Coalescing C
Rearranging
(4) Small C fragments around Ni catalyst
(5) Ni encapsulating with regular graphitic layer
Graphitization
C further deposition
(6) Ni encapsulation with discontinuous layers on surface

Figure S4: The growth mechanism of carbon produced by gas phase nanocatalytic routes for CS formation (A) and the role of catalysts in gas phase CS synthesis. Figures are adapted with permission from [1] and [2].

References

1 Xu, B.-s. Prospects and research progress in nano onion-like fullerenes. *New carbon materials* **23**, 289-301 (2008).

2 Wang, Z. & Kang, Z. Pairing of pentagonal and heptagonal carbon rings in the growth of nanosize carbon spheres synthesized by a mixed-valent oxide-catalytic carbonization process. *The Journal of Physical Chemistry* **100**, 17725-17731 (1996).
